# Mechanisms of Leukemia Immune Evasion and Their Role in Relapse After Haploidentical Hematopoietic Cell Transplantation

**DOI:** 10.3389/fimmu.2020.00147

**Published:** 2020-02-25

**Authors:** Pier Edoardo Rovatti, Valentina Gambacorta, Francesca Lorentino, Fabio Ciceri, Luca Vago

**Affiliations:** ^1^Unit of Immunogenetics, Leukemia Genomics and Immunobiology, IRCCS San Raffaele Scientific Institute, Milan, Italy; ^2^Hematology and Bone Marrow Transplantation Unit, IRCCS San Raffaele Scientific Institute, Milan, Italy; ^3^Unit of Senescence in Stem Cell Aging, Differentiation and Cancer, San Raffaele Telethon Institute for Gene Therapy, IRCCS San Raffaele Scientific Institute, Milan, Italy; ^4^Vita-Salute San Raffaele University, Milan, Italy

**Keywords:** haploidentical allogeneic hematopoietic stem cell transplantation, relapse, immune escape, HLA, immune check point

## Abstract

Over the last decade, the development of multiple strategies to allow the safe transfer from the donor to the patient of high numbers of partially HLA-incompatible T cells has dramatically reduced the toxicities of haploidentical hematopoietic cell transplantation (haplo-HCT), but this was not accompanied by a similar positive impact on the incidence of post-transplantation relapse. In the present review, we will elaborate on how the unique interplay between HLA-mismatched immune system and malignancy that characterizes haplo-HCT may impact relapse biology, shaping the selection of disease variants that are resistant to the “graft-vs.-leukemia” effect. In particular, we will present current knowledge on genomic loss of HLA, a relapse modality first described in haplo-HCT and accounting for a significant proportion of relapses in this setting, and discuss other more recently identified mechanisms of post-transplantation immune evasion and relapse, including the transcriptional downregulation of HLA class II molecules and the enforcement of inhibitory checkpoints between T cells and leukemia. Ultimately, we will review the available treatment options for patients who relapse after haplo-HCT and discuss on how a deeper insight into relapse immunobiology might inform the rational and personalized selection of therapies to improve the largely unsatisfactory clinical outcome of relapsing patients.

## Introduction

Allogeneic hematopoietic cell transplantation from haploidentical family members represents a promising solution to offer allogeneic HCT to virtually all patients with an indication to transplant, but lacking a fully compatible and/or rapidly available donor. However, from the immunological standpoint, haplo-HCT also represents the most challenging transplantation setting, counterpoising two largely HLA-incompatible immune systems and thus posing a severe risk of graft-vs.-host disease (GvHD) and immune rejection. To overcome this obstacle, over the last few decades, many strategies have been developed to improve the feasibility and safety of haplo-HCT (1, 2). In particular, two main haplo-HCT “philosophies” were progressively refined over the years: the *ex vivo* manipulation of the graft to deplete the most alloreactive cell subsets (3), eventually reinfusing them in a subsequent moment in combination with regulatory T cells (4, 5) or upon incorporation of safety switches (6–8), vs. the infusion of unmanipulated grafts, followed by administration of drugs capable of eliminating alloreactive cells *in vivo* (9, 10). Noticeably, some of these platforms have demonstrated remarkable success, leading to an exponential increase in the number of haplo-HCT performed worldwide (11, 12).

The development of innovative strategies to render haplo-HCT feasible was fueled by intensive research on the immunobiology of allo-HCT, leading to a number of observations that were later extended to other transplantation settings or even served as the foundation to explain the physiological metrics of immune responses to pathogens and tumors.

In the present review, we will present one of the most paradigmatic examples of this process by describing how investigation of mechanisms of relapse after haplo-HCT paved the way to understanding the interplay between transplanted immune system and tumor also in other transplantation settings and, importantly, to the development of new rationales for relapse therapy.

## Tumor-Intrinsic Mechanisms of Relapse

**Figure 1:**
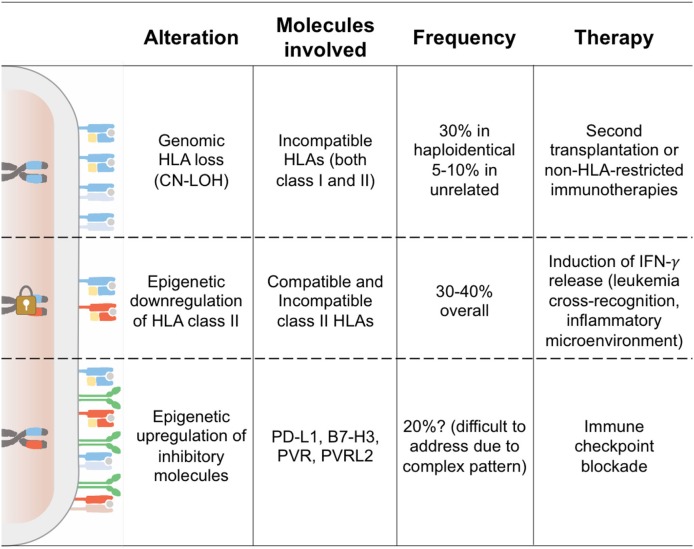
Tumor-Intrinsic Mechanisms of Immune Evasion and Relapse. This cartoon summarizes the features of the three modalities of leukemia immune evasion and relapse after allo-HCT better characterized to date. Chromosomes indicate the HLA haplotype homo- or hetero-zygosity, showing in cyan the donor-recipient shared haplotype and in red the patient-specific incompatible haplotype. The padlock symbolizes epigenetic silencing of the HLA class II loci. On the cell surface, HLA class I molecules are shown as heterodimers of HLA and beta-2-microglobulin (in yellow), HLA class II as dimers of two transmembrane single-chain HLA molecules, and inhibitory ligands as green homodimers.

Seminal studies conducted by the Seattle group more than 25 years ago led to the identification of donor-derived T cells as one of the major drivers of the graft-vs.-leukemia (GvL) effect (13). It is thus no surprise that all the best-characterized tumor-intrinsic mechanisms of immune evasion and relapse after allo-HCT have as a final output the abrogation of interactions between T cells and the tumor. This can occur either because leukemia cells become “invisible” to patrolling T cells, for instance through genetic or epigenetic alterations in the antigen processing and presenting machinery, or because they enact mechanisms to render the encounter ineffectual, as when inhibitory immune checkpoints are enforced (Figure 1).

### Genomic Loss of HLA

Alterations in the expression and functionality of HLA class I and II molecules have long been characterized in solid tumors, underlining also in this setting the importance of T cell-mediated responses in shaping tumor immunogenicity (14).

Interestingly, in hematological tumors, and acute myeloid leukemia (AML) in particular, alterations in the HLA region are quite uncommon, especially at the time of diagnosis (15, 16). This feature is critical, since the donor T cell-mediated GvL effect of allo-HCT mostly depends on the HLA molecule expression on the surface of leukemic cells. As part of the antigen-presenting machinery, HLA molecules serve as restriction elements for minor histocompatibility antigens and tumor-associated antigens or, when incompatible, as direct targets of primary alloreactivity. In haplo-HCT especially, where an entire HLA haplotype is mismatched between patient and donor, T cell-mediated alloreactivity converges against the incompatible molecules that rapidly become the immunodominant GvL targets.

Given this fundamental role of HLAs in the biology of haplo-HCT, it is reasonable that a possible getaway for malignant cells to escape the bottleneck of immunological pressure might be to exploit alterations in the HLA locus, mirroring what happens in solid tumors.

The first characterization of such a strategy being used in AML after haplo-HCT was provided nearly 10 years ago, when genomic loss of the mismatched HLA haplotype (from this point on referred to as “HLA loss”) was first reported (17). Behind this discovery, there is a curious case of serendipity: While investigating intermediate-resolution genomic HLA typing of bone marrow aspirate samples as an alternative technique for the assessment of hematopoietic chimerism (18), our group encountered several cases of AML post-transplantation relapse that typed negative for the patient HLAs. Genomic HLA typing of leukemic blasts purified from these relapses confirmed the absence of all HLA class I and class II genes encoded on the mismatched patient-specific HLA haplotype. A deeper examination of this phenomenon was then carried out exploiting whole-genome single-nucleotide polymorphism (SNP) arrays, demonstrating loss of heterozygosity (LOH) of chromosome 6p in the absence of copy number variations (CNVs), thus suggesting an event of acquired somatic uniparental disomy (aUPD). UPD has been described as a common chromosomal aberration in different tumor types, both solid and hematological (19–21). This genomic alteration consists of the loss of a chromosome region that is subsequently replaced by the homologous copy, resulting in acquired homozygosity of that region without the actual loss of genomic material. The consequences of this event can be diverse: We can witness an increase in the expression of oncogenes, loss of heterozygosity of mutated tumor-suppressors, or in this specific context, the loss of the HLA molecules not shared between donor and recipient, which represented the most immunodominant targets for donor T cell alloreactivity. In the case of HLA loss, the observed rearrangements had variable boundaries and extension in the different patients, but in most cases, encompassed the entire HLA region and, therefore, included all HLA class I and class II loci.

*Ex vivo* coculture of donor T cells with leukemic cells demonstrated that when HLA loss occurs, mutated blasts become completely invisible to donor T cells that were capable of recognizing them before transplantation, thus taking the upper hand over other clones and rapidly becoming the predominant population (17, 22). Documentation of HLA loss not only provides an explanation for how disease escaped a pre-existing control, but also contraindicates the infusion of additional donor T cells as a strategy to try to revert relapse, since also these cells would fail to find a target to attack.

Conversely, HLA loss variants that become invisible to donor T cell allorecognition could in principle still represent viable targets for alloreactive donor natural killer (NK) cells. Indeed, while the mechanism of aUPD does not reduce the overall surface levels of HLA class I molecules on the leukemia cell surface, thus avoiding to trigger “missing self” recognition by NK cells (23), the HLA alleles that are lost by leukemia cells often also represent ligands for donor inhibitory KIRs (24). Nonetheless, HLA loss relapses still occur, and the biology at the basis of NK cell failure in preventing or controlling the emergence of HLA loss relapses needs to be investigated further. This is highly relevant from the translational standpoint, since an improved understanding of NK cell responses in the context of HLA loss could also serve as a springboard to design adoptive immunotherapy trials based on NK cells to treat, or even prevent, these relapse variants.

One of the most relevant open issues regarding HLA loss is understanding when the genetic alteration occurs, or in other terms, if an infinitesimally small immune-resistant clone exists before allo-HCT or not. To date, the molecular drivers of aUPD are poorly known. It has been demonstrated that an increased susceptibility for chromosomal breaks and the effects of DNA damage inducers, including chemotherapeutic agents, might lead to higher aUPD risk in tight proximity of mitotic recombination sites (20), jeopardizing those heavily treated patients who undergo the transplantation procedure after multiple lines of chemotherapy. However, there is also evidence that aUPD can also be a common finding in AML samples at the time of diagnosis, with a large study on 454 samples reporting aUPD frequency of 15–20%. This alteration mainly affects specific chromosome arms, including 13q, 11p, and 11q (25). Of note in these reports, the involvement of the HLA region located in chromosome 6p is exceptional, with an estimated 3–4% of myeloid malignancies characterized by HLA LOH at disease onset (26, 27).

Some suggestions on the biological origin of HLA loss relapses come from retrospective clinical studies. In the largest analysis on this topic performed to date (28), HLA loss relapses were shown to occur significantly later than their “classical” counterparts and to be strongly associated to allo-HCTs performed in an active disease stage. A possible explanation linking these two observations might be that patients transplanted with a sizable leukemia burden probably present also much higher intratumoral heterogeneity than those transplanted with minimal or even undetectable residual disease and are thus also more likely to carry a clone with HLA loss or with high predisposition to aUPD, which may then slowly but steadily grow in the subsequent months following transplantation.

Soon after the initial description (17), a number of other studies reported cases of HLA loss relapses after haplo-HCT, with an incidence ranging between 20 and 40% of all relapses occurring in this setting (22, 29, 30). Of interest, analysis of two different cohorts transplanted at our institution using the same haplo-HCT backbone and differing only in the use of anti-thymocyte globulin (31) or high-dose cyclophosphamide (32) as *in vivo* T cell-depleting agent showed superimposable frequency of HLA loss relapses, suggesting that regardless of the strategy used, a significant population of alloreactive T cells escapes the initial purging and is capable to mediate significant antileukemic immune pressure. Studies specifically focused on T cell-depleted haplo-HCTs are to date lacking, but available data from T cell replete platforms indicate that the frequency of HLA loss is directly associated to the number of T cells transferred as part of the graft or after that (28), thus suggesting that in “T cell naked” transplants, HLA loss might be a rare event and relapses might have different underlying biology.

Interestingly, there have been reports of HLA loss relapses occurring in other transplantation settings, in particular after mismatched unrelated donor HCT (33–36) and, less frequently, after matched unrelated donor HCT (36). Although these reports originate from small cohorts of patients and therefore cannot provide an accurate estimate of the actual incidence of HLA loss in these settings, they appear to indicate a lower incidence of the phenomenon as compared to haplo-HCT. We can speculate that this lower frequency might indicate that, when donor-recipient incompatibilities are fewer, T cell alloreactivity and GvL effect is less pronouncedly focused against incompatible HLAs and possibly outperformed by immunodominant minor histocompatibility antigens. Moreover, it should be considered that in the unrelated HCT setting, the incompatibilities are often not *in cis* on the same haplotype, meaning that losing one haplotype by aUPD might not be as effective in abrogating immune pressure as in the haplo-HCT setting. Finally, the relative contribution of mismatches at the different HLA loci in driving alloreactivity and the GvL effect is not entirely clear, and it may be possible that some, but not other, incompatibilities might be more potent in promoting immune escape by HLA loss. In conclusion, more studies regarding the characterization of relapses outside of the haploidentical setting are still needed, and the complete understanding of how the GvL effect and the strength of the selective pressure mediated by alloreactive T-cells influences and shapes the underlying biological mechanisms of relapse in these contexts is yet to be entirely dissected.

It has already been stated how the occurrence of this genomic alteration greatly impairs T cell allorecognition, prompting the need for a more personalized clinical management of these relapses. As a consequence, the acute leukemia working party (ALWP) of the European Society for Blood and Marrow Transplantation (EBMT) recently made recommendations for testing eventual HLA loss at the time of relapse before employing donor lymphocyte infusions (DLIs) (37). However, until recently, documentation of HLA loss at relapse required the presence of a considerable tumor burden to perform HLA typing of either unprocessed bone marrow samples or, when possible, sorted leukemic blasts (18). To overcome these limitations, we recently developed “HLA-KMR,” a rapid, reliable, and economic assay based on quantitative PCR (qPCR) (38) that almost immediately became a commercially available diagnostic tool (GenDx, The Netherlands). The rationale of HLA-KMRs is to combine the detection of non-HLA-polymorphisms together with *ad hoc* qPCR reactions targeting the most common HLA allele groups. Therefore, in “classical” relapses, non-HLA and patient-specific HLA markers are concordantly positive, whereas the absence of HLA-specific signal indicates HLA loss relapse. This tool provides a sensitive method to detect HLA loss relapses event at early stages, allowing fast clinical decision-making and the use of a personalized therapeutic approach for every patient.

Finally, what should be the most appropriate therapeutic approach for HLA loss relapses occurring after haplo-HCT? Taking into consideration the mechanism and immunological consequences of this genomic alteration, a possible strategy could be a second haploidentical transplantation from an alternative donor, selected to target the remaining HLA haplotype. This originates from a unique situation, where donor T cells still share one haplotype with non-hematopoietic tissues, while being fully mismatched with the leukemic blasts, possibly providing an even stronger GvL effect (39). As a proof of concept, this approach was the one associated with the longest survival after relapse for patients experiencing HLA loss at our center (28) and might explain the superior outcome described by Imus and collaborators upon choosing donors with a different HLA-haplotype for second haplo-HCT (40). Unfortunately, a second allo-HCT is often not feasible in elderly or heavily pretreated patients, prompting further preclinical and clinical studies to treat HLA loss relapses using non-HLA-restricted immunotherapy approaches, including bispecific antibodies and chimeric antigen receptor (CAR)-modified T cells.

### Downregulation of HLA Class II Molecules

Two very recent studies provided remarkable evidence that genomic haplotype loss is not the only strategy used by leukemic cells to alter their HLA assets and avoid detection by donor-derived T cells. In both studies, comparison of samples pairwise collected from patients before and after allo-HCT led to appreciate that in up to 40% of post-transplantation relapses, the surface expression of HLA class II molecules (HLA-DR, -DQ, and -DP) becomes virtually absent, and this translates to the failure of donor T cells primed against the original disease to recognize the relapse variants (41, 42). Supporting previous studies conducted in animal models (43), this evidence suggests that interactions between HLA class II molecules and CD4 T cells are necessary for a proficient GvL effect and that this non-redundant arm of the antitumor immunity represents a vulnerability that is easily exploited by leukemia to reemerge. It should be noted, however, that HLA class II expression is also emerging as a relevant prognostic parameter in a number of other malignancies. HLA class II negativity has in fact been linked to unfavorable outcome in patients diagnosed with germinal center B-cell like diffuse large B cell lymphoma (44, 45) and with microsatellite stable carcinomas (46). In a study performed on relapsed/refractory classical Hodgkin's lymphoma, in addition to the positivity for PD-L1, high surface expression of HLA class II molecules also correlated with a better response to the anti PD-1 monoclonal antibody nivolumab (47).

Coming back to leukemia post-transplantation relapses, similar to the previously described genomic HLA loss mechanism, in this case a higher dose of T cells infused with the graft is also associated with a higher likelihood of also experiencing this modality of relapse (41). However, different from haplotype loss, class II downregulation has to date been observed with similar frequencies in both HLA-compatible and incompatible transplants (41, 42). This observation suggests that the driver of this event might not be alloreactivity toward incompatible HLA class II molecules, but rather against their presented repertoire of tumor-specific antigens and minor histocompatibility antigens. Recently, *in silico* studies convincingly showed that the number of minor histocompatibility antigens that are presented by HLA class II molecules surpasses its HLA class I counterpart by more than one logarithm (48, 49), suggesting that in the unrelated donor setting, immune reactivity against minors might be even more potent than the one against the few incompatible HLA molecules.

Of note, in both studies that first described HLA class II downregulation as an immune escape modality, in-depth genetic profiling of the relapsed leukemia found no evidence of mutations in HLA genes or their regulators, arguing toward an epigenetic origin of the observed phenotype. Gene expression analysis, performed to assess the mechanism of HLA class II expression defects, revealed a significant downregulation of the major histocompatibility class II transactivator CIITA (*MHC2TA*) (41, 42), which in some patients was linked to hypermethylation of its promoters (42). We further showed that this feature is stably maintained upon transplantation and serial passages in immune-compromised mice, with levels of surface expression of HLA class II molecules in patient-derived xenografts (PDXs) perfectly mirroring those observed in the corresponding primary human samples (41).

Unexpectedly, however, when we infused donor-derived T cells to animals harboring the HLA class II-expressing diagnosis or the HLA class II-defective relapse, we observed that, although with a slower kinetics, the latter was also eventually recognized and eradicated. An in-depth study of this phenomenon showed that cross-recognition of murine antigens by the infused T cells led to the release of high levels of interferon-γ (IFN-γ) in the animal plasma and that this was followed by the recovery of HLA class II expression on leukemic cells (41). These findings were also confirmed by *ex vivo* experiments, in which post-transplantation leukemic blasts exposed to recombinant human IFN-γ recovered HLA class II expression, and this in turn reconvened donor T cell-mediated recognition (41, 42). From a translational perspective, these results imply that a proinflammatory environment, driven by GvHD or recognition of antigens presented by HLA class I molecules, might actually revert this mechanism of relapse and re-establish a proficient antileukemic response.

Whereas, the description of deregulated HLA class II expression as a mechanism of AML post-transplantation relapse is extremely recent, there are a number of other malignancies in which alterations in HLA class II have been extensively investigated and that might provide precious hints on the molecular driver of the phenomenon in AML. For instance, there have been several reports of HLA class II downregulation in lymphoma cells as a consequence of deletions and point mutations of HLA class II genes and their regulators, including CIITA (44, 45). Moreover, in lymphomas, CIITA has been reported to be a recurrent fusion partner of the programmed death-ligands *CD274/PD-L1* and *CD273/PD-L2*, leading to the downregulation of HLA class II genes and the upregulation of PD-L1 and PD-L2 (50). In addition, loss of HLA class II expression has also been linked to epigenetic silencing, as a consequence of mutations in epigenetic regulators (e.g., enhancer of zeste homolog 2, *EZH2*) or of hypermethylation or hypoacetylation of the promoters of HLA genes and/or CIITA (42, 44, 45, 51)_._ Finally, Tarafdar et al. also proposed a cytokine-mediated pathway of HLA class II silencing active in chronic myeloid leukemia: In this disease, tumor cells can produce anti-inflammatory cytokines including IL-4 (52) and TGF-β (53) that downregulate the expression of CIITA, rendering themselves less immunogenic and susceptible to T cell recognition (52, 54).

### Upregulation of T Cell Inhibitory Ligands

While genomic and epigenetic alterations in HLA genes have all the final effect of turning tumor cells invisible to the donor-derived immune system, there is emerging evidence that leukemic cells can also hide in plain sight, using their encounter with T cells to transmit inhibitory signals that stun and impair antigen-specific responses. A number of reports have in fact shown that over the course of treatments and in particular after allo-HCT, hematologic malignancies increase their expression of molecules that inhibit T cell responses or drive their exhaustion, including members of the programmed death-ligand family (41, 55). In a recent study, retrospectively analyzing samples pairwise collected from AML patients at the time of diagnosis and at post-transplantation relapse, we showed increased expression of the inhibitory molecules PD-L1, CD276/B7-H3, and CD155/PVRL2 in up to 40% of cases of relapse. PD-L1 overexpression on AML blasts impaired donor T cell functions *ex vivo*, and antileukemic responses could be partially restored upon treatment with anti-PD-L1 monoclonal antibody (41). It should be noted, however, that in most patients, the landscape of expression of inhibitory ligands at time of relapse was quite composite, with high inter-patient variability, hinting at the fact that blocking a single interaction might yield limited clinical benefits and that efforts should rather be aimed at identifying and targeting a shared regulator of these molecules. The relative frequency of changes in T cell costimulation molecules remained superimposable when analyzed in different cohorts of patients receiving allo-HCT from donors with variable levels of HLA-compatibility (37), similarly to patients experiencing downregulation of HLA class II molecules at relapse and differently from patients with genomic loss of HLA-haplotype.

However, to date, little is known about the molecular drivers of this phenotype in the post-transplantation setting, and most of the currently available knowledge relates to PD-L1 and its regulation in other malignancies. Activation of aberrant janus kinase (JAK) signaling through 9p24.1 amplification has been shown to be a potent driver of PD-L1 upregulation in Hodgkin's lymphoma (56). Also, myeloproliferative neoplasms bearing the *JAK*^*V*617*F*^ point mutation had the same effect on PD-L1 expression (57). With that said, loss-of-function mutations in the JAK/STAT pathway observed in several other tumor types (e.g., melanoma) have been proven to be associated with resistance to PD-1/PD-L1 blockade (58–60). Also, Myc-driven lymphomas display constitutive upregulation of inhibitory molecules: Myc oncogenic signaling has been shown in fact to increase the expression of PD-L1 and of the “don't eat me” signal CD47 in tumor cells, impairing interactions with T lymphocytes and dendritic cells (61). Beside oncogenes driving PD-L1 overexpression, several epigenetic mechanisms have also been reported. Expression of PD-L1 has been shown, for instance, to be inversely correlated with methylation of its promoter and robustly induced upon treatment of tumor cells with hypomethylating agents (62). Also, micro RNAs (miRNAs), have been implicated in the regulation of PD-L1 expression by binding to the PD-L1 mRNA and driving its degradation; in AML, for instance, the levels of miRNA-34a showed inverse correlation with PD-L1 expression (63). Another emerging layer of regulation of PD-L1 is represented by post-translational modifications—for instance, through glycosylation of the mature protein (64).

In addition to all the tumor-intrinsic mechanisms of PD-L1 regulation mentioned in the previous paragraph, pro-inflammatory molecules (e.g., IFN-γ) secreted in the tumor microenvironment can also potently drive upregulation of PD-L1 on tumor cells (65). This might be extremely relevant in the setting of leukemia post-transplantation relapses, since, as discussed in the previous section, induction of a pro-inflammatory microenvironment conversely represents the key to reverting epigenetic downregulation of HLA class II molecules. Indeed, when data regarding expression of HLA molecules and inhibitory ligands at relapse in our patient cohorts were plotted together, it appeared quite evident that these two modalities of relapse are largely non-overlapping (41) and should prospectively be discriminated one from the other to enact the most appropriate salvage treatments.

Noticeably, the phenotypic features of T cells circulating in patients at the time of relapse mirror the changes observed in leukemic cells, with significant upregulation of inhibitory receptors in the patients whose leukemias express the respective ligands (41, 66). Recent studies showed that expression of inhibitory receptors such as PD-1 on T lymphocytes can at least in part be prompted by the intense stimulation conveyed to the donor immune system upon transfer into an allogeneic environment (67), as suggested also by the observation of higher expression of inhibitory receptors on the T cells of patients who received haplo-HCTs (66). However, in-depth analysis of T cells from patients who did or did not experience relapse allowed for the identification of specific exhaustion features in T cells from relapsing patients, with co-expression of multiple inhibitory receptors not only in terminally differentiated effectors, but also in early-differentiated memory stem and central memory T cells (66, 68). The exhausted phenotype was particularly evident in the patients' bone marrow, where T cell-leukemia interactions are mainly expected to occur and associated with a skewed T cell receptor (TCR) repertoire (66). Importantly, backtracking the clinical follow-up of patients who eventually relapsed, it was possible to identify the T cell exhaustion signature even months before relapse (41, 66) and in patients who relapsed after sole chemotherapy (69), suggesting that, upon further validation, these features might be used as an indicator to guide pre-emptive therapeutic approaches.

## Tumor-Extrinsic Mechanisms of Relapse

**Figure 2:**
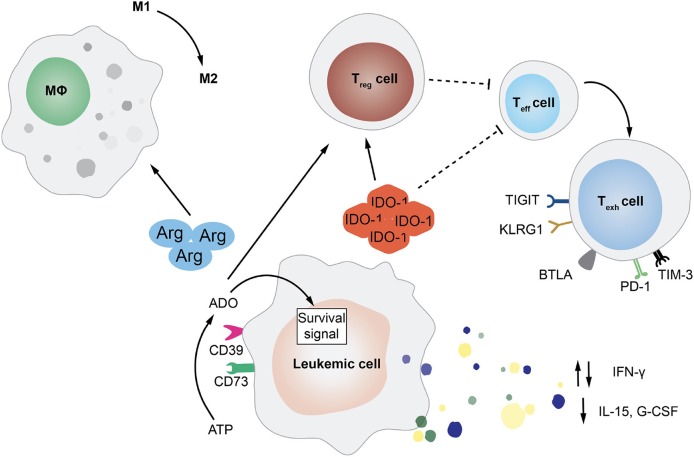
Tumor-Extrinsic Mechanisms of Immune Evasion and Relapse. This cartoon summarizes several of the pathways exploited by leukemic cells in order to rewire the bone marrow microenvironment and evade immune recognition. In particular, featured in the figure are the deregulated release by AML blasts of cytokines, such as interferon-γ (IFN-γ), interleukin-15 (IL-15), and granulocyte-colony stimulating factor (G-CSF); the expression of enzymes involved in aminoacid metabolism, such as arginase (Arg) and indoleamine 2,3-dioxygenase (IDO-1); and the upregulation of the ectonucleotidases CD73 and CD39 that leads to the increase in extracellular adenosine (ADO). All of these mediators can have an impact on the frequency and function of immune cell subsets, impairing T and NK cell activity, driving effector T cells toward exhaustion, inducing the expansion of regulatory T cells (Treg), and promoting the phenotypic switch of macrophages from pro-inflammatory M1 to immuno-suppressive M2.

Beside altering their features to increase aggressiveness and reduce immunogenicity, malignant cells can also accelerate disease progression by rewiring the microenvironment to their advantage, coopting the niche and the physiological mechanisms at the basis of immune tolerance. Mostly investigated in the context of solid tumors, interactions between cancer cells and the microenvironment are also starting to gain more attention in hematological diseases and gain an additional layer of complexity upon allo-HCT, when the niche becomes an admixture of pathological and non-pathological elements of both host and donor origin (Figure 2).

One of the best characterized modalities employed by hematological tumors to alter the immune microenvironment that surrounds them is switching from the production of pro-inflammatory cytokines to the release of immunosuppressive molecules, including IL-10 and TGF-β. For instance, it has been shown that during transformation, myeloid cells can reduce their production of granulocyte colony-stimulating factor (G-CSF), IL-15, and IFN-γ. Defects in IFN-γ production have been correlated to a specific polymorphism, which has been also linked to clinical risk parameters (e.g., prednisone response) in patients affected by B-lineage acute lymphocytic leukemia (ALL) (70). Strongly produced by normal myeloid progenitors, the physiological function of IL-15 is to expand and activate effector T and NK cells (71) and to promote the generation of memory stem T cell subset (72). Therefore, it is not difficult to understand why high levels of this cytokine in the tumor microenvironment are unfavorable for leukemic cells. In the post-transplantation setting, low plasma levels of IL-15 have been correlated to higher risk of relapse in patient affected by different hematological malignancies (73). One recently discovered mechanism at the basis of the reduced production of IL-15 by AML cells is the internal tandem duplication (ITD) of the FLT3 tyrosine kinase (FLT3-ITD) (74).

Even though in non-transplantation setting, the dysregulated effect of several metabolites has been shown to mediate immune suppression. The expression of indoleamine 2,3-dioxygenase-1 (IDO1) by leukemia cells was for instance correlated with unfavorable prognosis in childhood AML (75). IDO1 is the first actor of an enzymatic cascade resulting in the inhibition of T cell function and the T regulatory cell reprogramming (76). Moreover, AML exhibits the ability to block T cell function through the amino acid arginase, which can also drive macrophages toward the suppressive M2-like phenotype (77). Other two enzymes that are gaining recent attention for their possible role in inducing leukemia immune escape are the ectonucleotidase CD73 (78) and the ectonucleoside triphosphate diphosphohydrolase-1 CD39 (79).

Recently, moreover, studies conducted in solid tumors highlighted a major role of tumor-induced metabolic remodeling in altering T cell state and function. Specifically, Vodnala et al. revealed that the elevated presence of extracellular potassium in the tumor microenvironment can promote a state of functional starvation in tumor-specific T cells. The starvation response results in induction of autophagy and in epigenetic reprogramming, impairing T cell differentiation and function (80).

## Translating Relapse Biology Into Rationales for Treatment

The ideal strategies to treat relapse after allo-HCT should exert both a direct anti-tumor activity and enhance the alloreactive GvL effect of allogeneic T cells, sparing the risks of inducing significant cytopenias, immunosuppression, or GvHD. Moreover, considering the numerous and complexly combined modalities of relapse that were summarized in previous section, it should be considered that, ideally only using combinatorial therapies, it might be possible to hit a target without exposing the flank to compensatory responses that ultimately select alternative mechanisms of escape.

Here, we summarize the most recent evidence about post-HCT relapse treatment modalities, categorizing strategies that rely on cellular therapies or that aim at boosting or redirecting the pre-existent donor-derived immune system.

### Cellular Therapies

#### Donor Lymphocyte Infusions

One of the simplest and most intuitive ways to induce a GvL response after allogeneic HCT is to administer donor-lymphocyte infusions (DLIs). The main advantage of this strategy is the induction of a polyclonal T cell response able to target multiple antigens on malignant cells, reducing the risks of escaping T cell recognition just by loss of a single antigen. The major drawback of DLIs is represented by the possibility of donor T cells recognizing and attacking non-hematopoietic tissues, with the risk of triggering GvHD, which can turn out to be a serious, and often fatal, complication. This hazard can be significantly reduced either by incorporating suicide genes in the infused cells, acting as a “safety switch” in case of unwanted reactions (81, 82), or by infusing specific T cell subsets endowed with lower intrinsic alloreactivity, such as memory (83, 84) or γδ (85, 86) T cells.

As discussed in previous sections, when considering the therapeutic use of DLI for relapses after haplo-HCT, it is fundamental to rapidly determine if relapse is sustained by HLA loss immune-escape leukemia variants that represent a clear counter indication to DLI administration. In fact, the genomic loss of the unshared HLA haplotype in leukemia cells not only renders them invisible to the major HLA alloreactivity exerted by infused T cells, but it also does not impact their recognition of healthy tissues, leaving the risk of DLI-induced GvHD largely unaltered. For these reasons, upon documentation of HLA loss, other salvage options should be prioritized as treatment strategies (17, 39).

Outside of this specific context, a considerable body of literature exists on the use of DLI as therapy of relapse after haplo-HCT. The first study after unmanipulated haplo-HCT performed under ATG-based GvHD prophylaxis utilized a median dose of donor T cells of 0.6 × 10^8^ CD3^+^/Kg, reporting significant risks of both severe acute GvHD (aGvHD, 30%) and chronic GvHD (cGvHD, 64%) (87). More recently, a study testing DLI after post-transplant cyclophosphamide (PTCy)-based GvHD prophylaxis yielded a 30% complete remission (CR) rate, with a risk of developing grade III–IV aGvHD or cGvHD of 15 and 8%, respectively. In this trial, a dose of 1 × 10^6^ CD3^+^/Kg was considered a reasonable starting dose (88). Another study showed that escalating doses of DLI after PTCy-based haplo-HCT were accompanied by at least a 33% CR rate, a 14% risk of grade II–III aGvHD, and no cases of grade III–IV aGvHD or cGvHD. In this study, the initial administered DLI dose was 1 × 10^5^ CD3^+^/Kg, in case of molecular relapse, and higher (from 1 × 10^6^ rising to 1 × 10^7^ CD3^+^/Kg), in case of hematological relapse (89).

Several studies also reported results from combinatorial administration of DLI and immunomodulating agents, with the aim of increasing the immunogenicity of tumor cells, rendering them more susceptible to DLI action. The diverse dose schedules and time points of infusions preclude a clear guideline, but most trials employed a starting dose of 1 × 10^5^ CD3^+^/Kg, eventually escalating in the absence of GvHD development (88, 90–98).

#### Second Allogeneic Transplantation

Second allogeneic transplantation (allo-HCT2) to treat relapse after the first allo-HCT has recently gained more popularity, thanks to the introduction of reduced-intensity conditioning and improvements of supportive therapies, which have significantly reduced toxicities after allo-HCT2, historically burdened by treatment-related mortality up to 40–50% (99, 100). As in the case of DLI, when considering HCT2 for relapse after HLA-mismatched HCT, it is mandatory to discriminate whether relapse after the first transplant was classical or HLA loss. Especially in the second case, as mentioned above, selecting a second haploidentical donor with a different HLA haplotype provided some very promising preliminary results (40). Unfortunately, for all the studies on this subject, the inevitable selection bias of patients fit to receive a second conditioning and further transplantation must be taken into account, and clinical decisions must balance individual patient comorbidities and alternative therapeutic strategies.

#### Adoptive Immunotherapy With Genetically Redirected Immune Cells

Over the last few years, a number of landmark studies have demonstrated the feasibility and efficacy of using gene therapy to redirect immune cells in a non-HLA-restricted fashion against antigens of choice. The most striking example is provided by chimeric antigen receptor (CAR) T cells, which are capable of binding to the surface antigen of choice without the need for TCR-HLA interactions, thus representing a promising therapeutic option for patients relapsing with HLA loss or HLA downregulation. Moreover, CAR potent synthetic co-stimulatory domains may bypass the effect of the immune-suppressive signals expressed by tumor cells or microenvironment (101, 102).

CAR T cells targeting CD19 are, to date, the best studied and have demonstrated significant activity in chemotherapy refractory CLL, B-cell lymphomas and B-ALL in the autologous setting (103–108). There is also growing evidence of the efficacy of donor-origin CD19 CAR T cells in patients relapsing after allo-HCT (102, 108–111) or even haplo-HCT (112, 113). In this scenario, the infusion of allogeneic CAR T cells could carry the theoretical risk of GvHD; however, incidence of this fearsome complication in early trials was quite low, and an elegant study in mouse models showed that the CAR-driven and TCR-driven signal actually adds up, accelerating exhaustion and limiting alloreactions (101). Still, a number of studies are focusing on the development of improved strategies to further enhance CAR T efficacy and persistence without risking to induce GvHD, such as by transducing recipient-derived donor T cells (113), by using genome editing approaches to knock out the endogenous TCR (101, 114), or by modifying with the CAR different immune cells, less prone to induce GvHD (85, 115, 116).

### Redirecting or Boosting the Donor-Derived Immune System

#### Bispecific Antibodies

Based on a principle similar to the one that guided the development of CAR T cells, bispecific antibodies can also enable redirection of immune cells toward malignant cells, forcing the formation of an immunological synapsis through the binding of an antigen expressed on effectors (such as CD3 on T cells or CD16 on NK cells), with one expressed by the tumor target (such as CD19 for lymphoid malignancies or CD33 for myeloid leukemias) (117, 118). This results in the release of cytotoxic granules in close proximity to target cells, with the ultimate step of apoptosis induction and elimination, also fueled by inflammatory cytokines production and antigen spreading mechanisms (119). This strategy could be another useful way to circumvent HLA-restriction of TCR, with the potential added value of being readily available off the shelf and taking advantage of cells that are already circulating in the patient and tolerized against his healthy tissues. However, other immune-evasion mechanisms (related to the induction of inhibitory checkpoint molecules and the production of immunosuppressive cytokines or metabolites), have been shown to rapidly emerge upon treatment with bispecifics, suggesting that full exploitation of the anti-tumoral activity of these promising molecules could pass by enhancing costimulatory pathways or blocking immune checkpoints (120–125).

#### Epigenetic Therapies

The two commercially available hypomethylating agents (HMAs), azacytidine (Aza) and decitabine (DAC), are frequently used for post-HCT relapse treatment in AML or myelodysplastic syndromes (MDS). HMAs indirectly inhibit DNA methyltransferases significantly altering DNA methylation patterns with consequent induction of cell cycle arrest, DNA damage accumulation, apoptosis, and differentiation (126–132). More recently, immune-related effects of hypomethylating agents have also been described. In particular, Aza stimulates antitumor immunity inducing the upregulation on leukemic cells of leukemia-associated and minor-histocompatibility antigens, including PRAME, MAGE-A, NY-ESO1, and HA-1 (129, 130, 133–136). Aza can also lead to increased HLA class-I and II expression and modulate tumor-immunogenicity through the upregulation on leukemic cell surface of costimulatory molecules, such as CD80, CD86, ULBP, and MIC-A (137). Among the reported effects, HMAs can induce the expression of important players involved in anti-viral responses, including IFN-γ and cytokines. Interestingly, Aza can also promote upregulation of endogenous retroviral elements on tumor cells, inducing a “viral mimicry” response that ultimately results in the induction of anti-tumor immunity (138–140). However, Aza can also act as a double-edged sword, since it can upregulate PD-1, PD-L1/L2, and CTLA-4 inhibitory pathways and induce the expansion of regulatory T cells (131), potentially hampering the intensity and duration of cytotoxic T cell responses and facilitating the tolerization and exhaustion of tumor-specific T cells (141, 142).

Due to their reported immune-related effects, HMAs have been frequently employed in combination with DLI. To date, we have data on more than 600 patients undergoing salvage regimens including Aza and DLI, reporting very variable results in terms of clinical outcome (143–148). Because of the heterogeneous results obtained so far and lack of consent on treatment schedules, two retrospective surveys have analyzed the correlates of efficacy of Aza+DLI combinations in more homogeneous cohorts, one facilitated by the German Cooperative Transplant Study Group (145) and the other by the EBMT (146). These studies reported that patients that benefitted the most from Aza+DLI combinatorial approach were those who presented low disease burden at the time of relapse (molecular relapse or <20% blasts in bone marrow) and those with a longer interval from allo-HCT to relapse. These variables can be adopted to predict treatment response through a score assignment (AZA relapse prognostic score: ARPS), even if an independent validation cohort is still lacking (146).

Histone acetylation is another epigenetic mechanism of immune regulation, balanced between the activity of histone acetyl-transferases (HATs) and histone deacetylases (HDACs). HDAC inhibitors, such as vorinostat and panobinostat, have been associated with the upregulation of major-histocompatibility and co-stimulatory molecules on AML cell surface through the induction of an open and readable structure of chromatin (149, 150). To date, two prospective phase I/II trials of post-HCT therapy with panobinostat for AML/MDS patients, alone or in combination with DAC and DLI, have been reported (151, 152).

#### Immune Checkpoint Blockade

Immune checkpoint inhibition through the administration of monoclonal antibodies that target the PD-1/PD-L1 and CTLA-4/B7 axes is emerging as an attractive strategy to enhance alloreactive T cell function and rewire the immunosuppressive milieu in which disease relapse often occurs (153–155). Clinical trials exploring the efficacy of immune checkpoint inhibitors after allo-HCT have shown some promise using the anti-CTLA4 antibody ipilimumab (156, 157) and more modest results using PD-1 inhibitors in diseases other than Hodgkin's lymphoma (158–161). Moreover, post-transplantation treatment with checkpoint inhibitors appears to be associated to a significant risk of severe and treatment-refractory GvHD and immune-related events (162).

However, as the balance of stimulatory and inhibitory signals determines the magnitude of immune responses against tumor cells, combining HMAs and immune-checkpoint blockade therapies may represent an interesting approach to release the “break” signal received by tumor-reactive immune cells (163–165). A phase II trial exploring the combination of the anti-PD1 monoclonal antibody Nivolumab and Aza in relapsed AML reported an overall response rate of 33% (166), and several ongoing trials are assessing the efficacy of HMAs and immune checkpoint inhibitor combinations, some of them recruiting also post-transplantation relapsed patients (NCT02890329, NCT02845297, NCT02996474, and NCT02397720).

#### Cytokine Therapies

The use of exogenous cytokines to boost or restore T cell- and NK cell-impaired effector functions have been object of intense investigation in cancer therapy and especially in the field of hematological malignancies. Interleukin 2 (IL2), IFN-α, and IL-15 are the best studied. IL-2 has been shown to stimulate the anti-tumor effect of lymphocytes, polarizing helper T cell responses toward type 1 and exerting both immune-enhancing and immune-suppressive activities (167–169). However, application of IL-2 monotherapy against AML has yielded very limited clinical benefit, both for the induction of regulatory T cells that impaired antileukemic activity and for the rapid drop in effector functions due to T cells terminal differentiation and exhaustion (170–173). IFN-α, however, exerts pleiotropic functions, since it has a direct antileukemic effect and also possesses immune-stimulatory properties, leading to dendritic cells stimulation, enhancement of NK-cell cytotoxicity, and sensitization of T cells to other inflammatory cytokines, such as IL-2 (174–177). Despite these theoretical premises, IFN-α failed to demonstrate significant activity as single agent in post-transplantation relapse (178–181). As previously described, IL-15 is a potent immunostimulatory cytokine, that potentiates both T and NK cell immune responses, promoting the generation and maintenance of high-avidity and long-lived CD8^+^ memory T cells. IL-15 also prevents activation-induced T cell death and does not induce the expansion of immunosuppressive regulatory T cells (72, 182–186). A phase I trial testing the IL-15 super-agonist complex ALT-803 in patients relapsing after allo-HCT showed a very promising response rate (19% of evaluable patients), correlated to the expansion of both NK and T cells (187). Recently, novel approaches to transfer high concentration of cytokines to the tumor site and reduce their systemic effects are emerging, including gene therapy “Trojan Horse” strategies (188) and the use of lipid nanoparticles to convey to the tumor site mRNAs encoding cytokines (189).

### Immune-Related Effects of Targeted Therapies

The growing armamentarium of targeted therapies is providing new evidence that, beside their direct effects, some of them can also promote antitumor immunity. A recent work testing the effect of the tyrosine-kinase inhibitor sorafenib in a mouse model of leukemia showed that the treatment increased the production of IL-15 by leukemic cells bearing FLT3-ITD. This resulted in enhanced CD8^+^ T cell effector function (via their increased metabolic capacity) and leukemia eradication. Mechanistically, sorafenib induced transcription of IL-15, acting by inhibition of the transcription factor ATF435 that in turn suppresses the IL-15 activator interferon regulatory factor 7 (IRF7) (74).

Another example of tyrosine-kinase inhibitor exerting “off-target” immune mechanisms is represented by imatinib, which is indicated in Philadelphia-positive (Ph+) leukemias, namely chronic myeloid leukemia (CML) and Ph+ acute lymphoblastic leukemia (ALL). Allogeneic HCT remains the only curative option for Ph+ ALL and advanced-phase CML, and there is general consensus about imatinib administration following HCT (190, 191). In addition to targeting Bcr/Abl1 and KIT oncogene products, imatinib modulates the proliferation, polarization, and functionality of different subsets of myeloid and lymphoid cells (192–195). This modulation can exert either inhibitory or stimulating immune effects. Among the inhibitory effects are the inhibition of dendritic cells expansion, resulting in less efficient priming of cytotoxic T cells (196–199), the polarization toward a M2-like anti-inflammatory phenotype of tumor-associated macrophages (200, 201), the reduction of effector-cytokine production by CD4^+^ T cells in response to TCR-signaling (202, 203), and the reduction of IgM-producing memory B-cell frequency (204–206). On the other hand, imatinib also has stimulating effects such as decreased expression of 2,3-IDO and consequent apoptosis in regulatory T cells (207, 208); reduction of myeloid-derived suppressor cells, thus restoring a T cell cytotoxic response (209–211); reduced secretion of VEGF with subsequent antiangiogenic effect (212, 213); polarization toward a higher Th1/Th2 ratio (214–216); and preferential expression of activating NK receptors (217).

## Conclusions and Perspectives

The landscape of allo-HCT, and haplo-HCT in particular, is rapidly changing, with multiple platforms able to achieve remarkable long-term outcome results. The reduced risk of treatment-related toxicities and mortality has also opened the possibility to implement innovative pharmacological or cellular therapies in the post-transplantation follow-up, transforming the perception of allo-HCT from that of a final consolidation therapy to a “platform” to build on. In this new scenario, it will be of utmost relevance to also associate to the analysis of clinical endpoints a detailed study on how changing the recipe of allo-HCT influences its immunobiology. For instance, understanding the relative contribution of each immune cell subset transferred as part of the graft in the induction of GvHD and protection against relapse will be fundamental to guide further improvements in “tailoring” graft composition and post-transplantation cell therapies, as convincingly suggested by a number of recent studies (218–220). It is now evident that the success or failure of transplantation is linked to our ability to take full advantage of the many features endowed in the immune system and to combine them with targeted therapies to hit as many tumor targets as possible, reducing the chances of selection of escape variants. Generation of new quantitative systems to map tumor immune targets, characterization of the tumor immune microenvironment by multi-omics single-cell technologies, and generation of more refined humanized mouse model to mirror allo-HCT all appear to be promising avenues in advancing knowledge on allo-HCT immunobiology and, ultimately, in generating new rationales to further improve clinical outcome.

## Author Contributions

PR, VG, and FL reviewed available literature and drafted the paper. FC and LV provided critical discussion and revised the manuscript draft.

### Conflict of Interest

LV received research funding from GenDx (Utrecht, The Netherlands) and Moderna Therapeutics (Cambridge, MA, USA). The remaining authors declare that the research was conducted in the absence of any commercial or financial relationships that could be construed as a potential conflict of interest.
